# Anoikis resistant mediated by FASN promoted growth and metastasis of osteosarcoma

**DOI:** 10.1038/s41419-019-1532-2

**Published:** 2019-04-01

**Authors:** Tianhao Sun, Xing Zhong, Honghai Song, Jiaming Liu, Jingao Li, Frankie Leung, William W. Lu, Zhi-Li Liu

**Affiliations:** 1 Department of Orthopedic Surgery, The First Affiliated Hospital of Nanchang University, Nanchang, 330006 China; 2 Department of Orthopaedics and Traumatology, Li Ka Shing Faculty of Medicine, The University of Hong Kong, Hong Kong SAR, China; 3 Division of Chemotherapy, Jiangxi Cancer Hospital, Nanchang, Jiangxi Province China; 4 Shenzhen Institutes of Advanced Technology, Chinese Academy of Science, Shenzhen, 518000 China

## Abstract

The pulmonary metastasis of osteosarcoma (OS) occurs commonly, which resulted from anoikis resistant (AR) of tumor cells as reported by previous studies, but the exact roles of AR in osteosarcoma were not fully studied. Our previous investigations showed fatty acid synthase (FASN) was relating to clinical features of patients with OS. In this study, we aim to explore the functions of FASN in the AR OS cells in vitro and in vivo and study the downstream effectors of FASN. In the present study, we used our established cell model to study the AR. We revealed that AR promoted cell proliferation and migration as determined by colony formation assay and transwell assay. In addition, AR assisted tumor growth in vivo. In the AR cells, the expression of FASN was higher. Thus, we constructed lentiviruses to silence or overexpress FASN in four cell lines to study functions of FASN. Silence of FASN reduced cell colonies and migration while overexpression of FASN increased colonies and migration in suspended cells. Loss of functions of FASN induced cell apoptosis in suspended OS cells while gain of function of FASN suppressed apoptosis as determined by flow cytometry. We found the levels of p-ERK1/2 and Bcl-xL declined when FASN was silenced while they increased when FASN was overexpressed. In addition, results showed that the levels of FASN and its potential related molecules (p-ERK1/2 and Bcl-xL) increased in 143B-AR and MG-63-AR cells. In vivo study showed that inhibition of FASN decreased pulmonary metastasis of OS. In conclusion, we showed that anoikis resistant and FASN as two interactional factors facilitated the progress of osteosarcoma.

## Introduction

Osteosarcoma (OS) happens in adolescents and its fatality rate is high. Pulmonary metastasis is the leading cause of death for patients with OS, the 5-year survival rate is only 17–23%^1^. The pulmonary metastasis of OS occurs so commonly but the exact mechanisms are not very clear. Given the cellular and molecular mechanisms of OS pulmonary metastasis would help to improve the survival time in patients with OS. As all malignant tumors, the metastasis of OS involves many processes, including invasion, migration, distant survival, and proliferation. During migration, the cells detach from the cell matrix and neighboring cells. After losing attachment of neighboring cells, cells usually undergo an apoptotic procedure known as “anoikis”, a form of cell death. This detachment-induced cell apoptosis (anoikis) is relating to tumor metastasis. Malignant tumor cells with the ability to survive and proliferate under detached conditions are termed as anoikis resistant (AR) cells. Tumor cells acquire AR to survive after detaching from the original sites and travel through the circulatory systems to disseminate. One important reason of the pulmonary metastasis might be anoikis resistant of tumor cells^2,3^. There were studies of mechanisms of osteosarcoma^4^, but the exact mechanism of metastasis and the relating molecules were still not fully reported. Therefore, elucidation of the molecular mechanisms of AR has potentially profound relevance for the therapy and management of OS.

In the processes of the AR of OS, lipid rafts play important roles. The biosynthesis of the lipid rafts needs palmitic acid, a final metabolic product of fatty acid synthase (FASN)^5^. During the synthesis of endogenous fatty acids, the key enzyme FASN was responsible for catalyzing the synthesis of long-chain fatty acids in mammals. Also, FASN is critical in sustaining the biological features of malignant tumor cells^6^. FASN is expressed at high levels in a variety of human tumors such as prostate cancer^7^. In fact, FASN has been studied as a candidate oncogene in cancer^8^ such as prostate cancer^9^, liver cancer^10^, and ovarian cancer^11^. Recently evidences showed that fatty acid metabolic pathways played a critical role in carcinogenesis^12^. Inhibition of FASN expression could suppress malignant tumor cell proliferation in vitro and in vivo in oral squamous cell carcinomas^13^, liver cancer^14^, and neurogenesis^15^. Therefore, FASN has been considered as a promising target for anticancer treatment and management. However, the molecular roles of FASN in osteosarcoma cells remain unclear and need to be further studied. Increasing evidences showed that FASN also contribute to colorectal cancer cell metastasis^16^. Our previous studies focus on the roles of FASN in osteosarcoma^17^. We revealed that the expression levels of FASN determined by immunohistochemistry were higher in the patients with lung metastasis compared with those without metastasis^18^, indicating that FASN might promote pulmonary metastasis. However, the molecular experimental mechanisms of FASN promoting metastasis in OS retain unclear. One of the most important reasons why lung metastasis is anoikis resistant^2^. Whether FASN assists lung metastasis of OS by enhancing the anoikis resistant and the detailed molecular and cellular mechanisms need to be elucidated.

Therefore, we assume that FASN may prevent anoikis and promote metastasis in OS cells. In the present study, we investigated the effects of AR in OS and the functions of FASN in AR cells in vitro and in vivo. We also explored the potential downstream effectors of FASN. The results revealed that increased FASN could mediate OS cell anoikis resistance and promoted its pulmonary metastasis. In the processes, FASN regulated the activity of ERK1/2/Bcl-xL signaling pathway.

## Results

### Anoikis resistant promoted cell proliferation, cell migration, and tumor growth

**Fig. 1:**
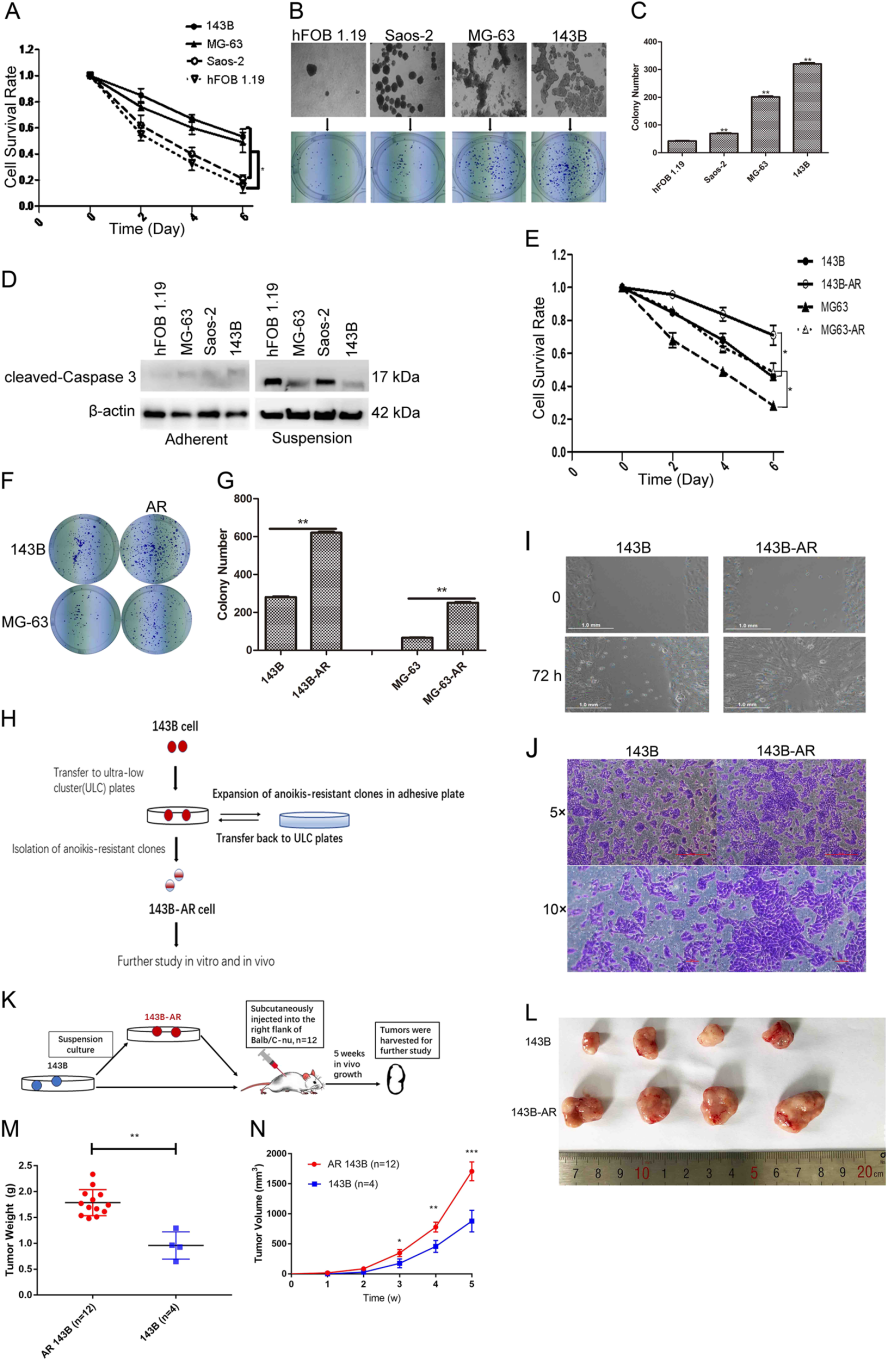
Anoikis resistant promoted cell proliferation, cell migration and tumor growth. **a** Cell survival rate of 143B, MG-63, Saos-2, and hFOB 1.19 after suspension for 0, 2, 4, and 6 days. **b** The four kinds of cell lines were suspended and taken photos by microscopy and then proceeded to colony formation assay. Representative pictures of colonies of cells (blue dots). **c** Quantitation of cell colony numbers. **d** Relative expression levels of cleaved-Caspase 3 in cells. β-actin was used as a loading control. **e** Cell survival rate of 143B, 143B-AR, MG-63, and MG-63-AR after suspension for 0, 2, 4, and 6 days. **f** Representative pictures of colonies of 143B, 143B-AR, MG-63, and MG-63-AR cells. **g** Quantitation of cell colony numbers. **h** Flow chart of generation of 143B-AR cells. **i** Wound healing assay. **j** Transwell cell migration assay. The cells were stained with crystal violet. **k** Flow chart of studying the AR effects in vivo. **l** Representative examples of tumors formed in nude mice injected with the indicated cells. **m** Tumor weight were summarized and compared in the chart. **n** Tumor growth curves were summarized in the line chart. The average tumor volume was expressed as the mean ± SD of mice. *p < 0.05, **p < 0.01, *n* = 3/group

Osteosarcoma cell lines Saos-2, MG-63, and 143B and non-tumor cell line hFOB 1.19 were all attached cells, so some cells would die when they were suspended culture. We suspended these four skeletal cell lines and then counted the survival cell number. We found that the cell survival rate from high to low was 143B, MG-63, Saos-2, and hFOB 1.19, respectively (Fig. 1a). From the microscopy we could found the Saos-2, MG-63, and 143B easily formed clusters while the hFOB 1.19 did not crowd together significantly (Fig. 1b). That might be one of the reasons why their survival rates were different. Colony formation assay also showed that the colony number of 143B and MG-63 was more than hFOB 1.19 (Fig. 1b, c). We then thought about the molecular mechanism of this phenomenon. We found the expression of cleaved-Caspase 3 of hFOB 1.19 and Saos-2 were higher after the cells were suspended (Fig. 1d), indicating that the cell apoptosis happened severely in these two cells. Thus, we suspended the 143B and MG-63 and then let them attach to flasks to screen anoikis resistant (AR) cells. As Fig. 1e showed this method were successful. We used colony formation assay and results also showed that there were more AR cells surviving compared with non-AR cells after all the cells were suspended (Fig. 1f, g). Because 143B had the best ability of AR, we then used this cell line for further study. To make better 143B-AR cells (Fig. 1h), we transferred common 143B cells into ultra-low cluster plates for days and then expanded of AR cells in adhesive flasks, and then transferred cells back to ultra-low cluster flasks. There are several returns of this procedure (Fig. 1h). Wound healing assay and transwell assay showed cell migration rate was higher in 143-AR cells than normal cells (Fig. 1i, j). We further verified these results in vivo (Fig. 1k). The tumor formation rate in mice was 100% (12/12) in the AR 143B treated mice compared with their control (143B) which was 33% (4/12). AR 143 cells resulted in bigger tumor size (Fig. 1l), higher tumor weight (Fig. 1m) and tumor volume (Fig. 1n) than non-AR cells.

### FASN was relating to anoikis resistance and confirmation of successful establishment of stable cell lines silencing or overexpressing FASN

**Fig. 2:**
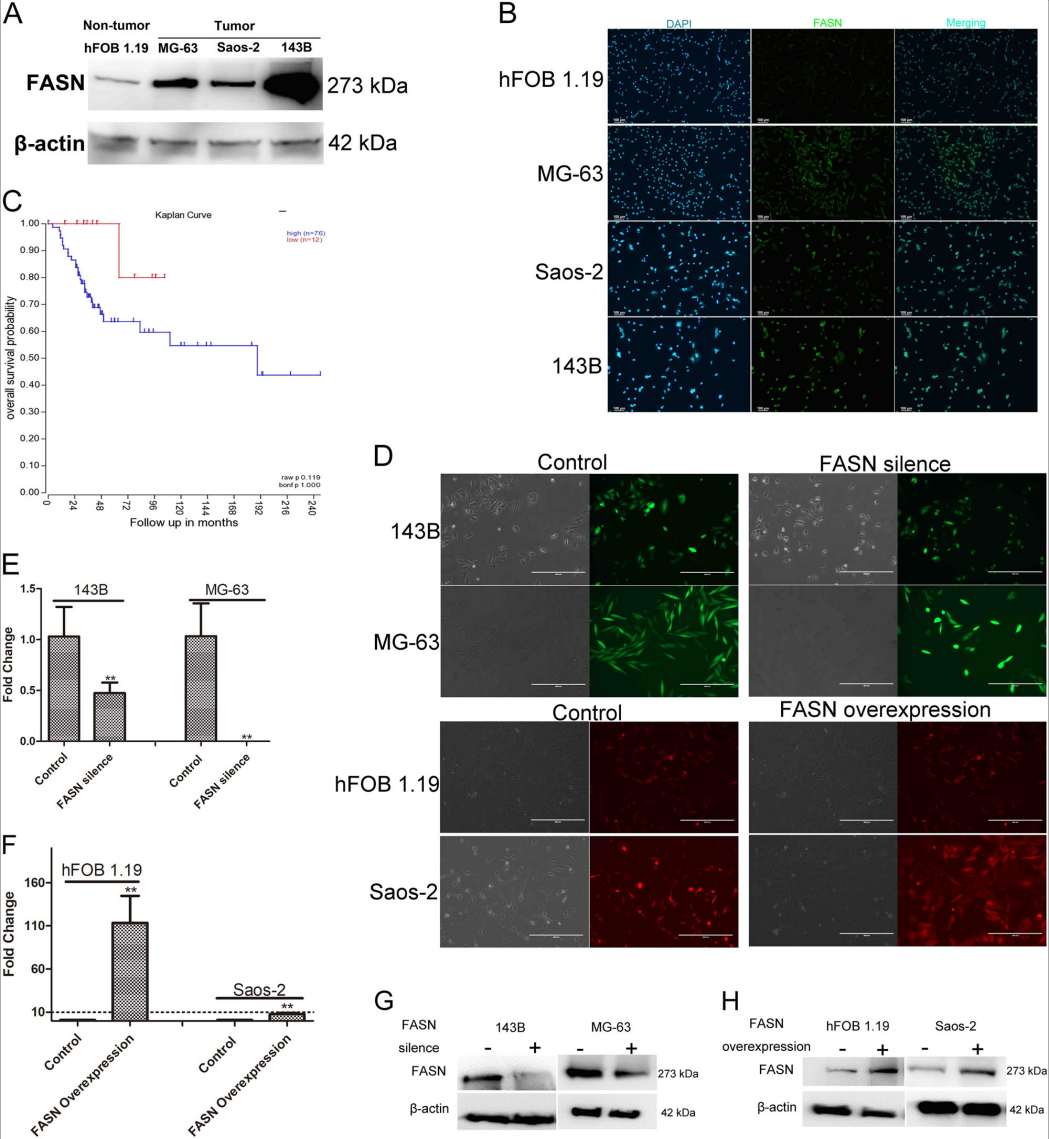
FASN was relating to anoikis resistance and confirmation of successful establishment of stable cell lines silencing or overexpressing FASN. **a** Relative expression levels of FASN in tumor and non-tumor cells. **b** Representative staining images of cells. The intensity and the degree of green represented the expression levels of FASN. The cells were greener in tumor cells than non-tumor cells. In other words, the degree of green was higher in the tumor cells than the controls. **c** Kaplan–Meier analysis of overall survival rate related to the expression of FASN in 88 OS cases based on a human osteosarcoma gene expression database (https://hgserver1.amc.nl/cgi-bin/r2/main.cgi). **d** The photography of the 143B and MG-63 cell lines infected with lentivirus controls (left) or lentivirus shFASN (right). The photography of the hFOB 1.19 and Saos-2 cell lines infected with lentivirus controls (left) or lentivirus containing FASN overexpression plasmids (right). **e** Relative expression levels of FASN in 143B and MG-63 cells treated with shFASN. **f** Relative expression levels of FASN in hFOB 1.19 and Saos-2 cells treated with lentivirus containing FASN overexpression plasmids. **g** The expression levels of FASN were compared between shFASN-treated cells and controls in 143B and MG-63 cells by western blot. **h** The expression levels of FASN were compared between FASN overexpression lentivirus-treated cells and controls in hFOB 1.19 and Saos-2 cells by western blot

Next, we explored the molecular mechanism of AR. In the present study, on the one hand, we found the cell survival rate of cells were different after suspension (Fig. 1a–c). Specifically, the survival rate of 143B was highest, followed by MG-63 and Saos-2, and the survival rate of hFOB 1.19 was lowest (Fig. 1a–c). Our previous studies showed that cell apoptosis might be relating to FASN^19^. On the other hand, in consistent with these results, the expression of FASN in 143B was highest, followed by MG-63 and Saos-2, and the expression of FASN in hFOB 1.19 was lowest as detected by western blot and ICC (Fig. 2a, b). In addition, we studied the clinical relevance of FASN. To further determine the prognostic significance of FASN expression in patients with OS, an online human OS gene expression database was used. Kaplan–Meier analysis revealed correlation between the expression of FASN and overall survival during the follow-up period, while patients with a higher expression of FASN had a significantly lower overall survival rate (Fig. 2c). The results indicated that FASN was upregulated in OS. A high expression of FASN predicted a poor overall survival rate and might facilitate the progression of OS. Last but not least, FASN was reported to be relating to cancer cell apoptosis^20^. Taken together, these two aspects of results indicated that there were chances that FASN was relating to anoikis resistance and clinic. Therefore, to further study the roles of FASN in suspended tumor cells, we used lentivirus method to infect the cells. We used total four cell lines and two (143B and MG-63) of them were used for silencing FASN by shRNA while the other two (hFOB 1.19 and Saos-2) of them were used for overexpressing FASN. The constructed plasmids contained GFP and mCherry, respectively, so the fluorescence in the transfected cells confirmed the success of transfection (Fig. 2d). Then we used qPCR, western blot and ICC to detect the expression of FASN. The mRNA levels of FASN were significantly downregulated by shFASN in 143B and MG-63 while they were upregulated substantially in hFOB 1.19 and Saos-2 cells (Fig. 2e, f). In addition, the protein levels of FASN were decreased by shFASN in both 143B and MG-63 (Fig. 2g) while the protein levels were increased by lentivirus containing FASN expressing plasmid (Fig. 2h). We then used ICC to confirm the results. Specifically, the degree of green fluorescence was lower in FASN silence group (Fig S1A and Fig S1B) while they were higher in FASN overexpression group (Fig S1C and Fig S2).

### FASN promoted cell proliferation, cell migration, and inhibited cell apoptosis in suspended cells

**Fig. 3:**
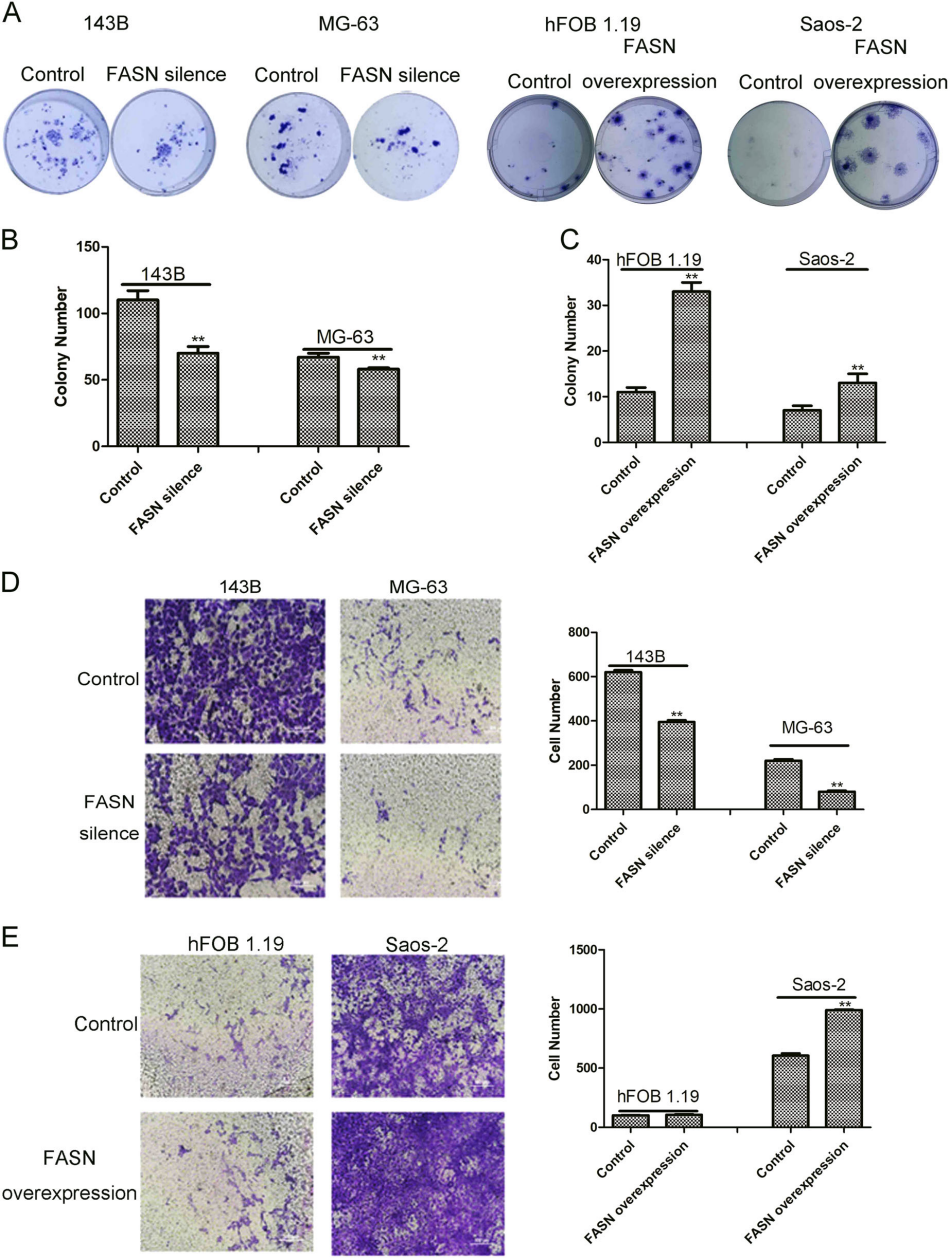
FASN promoted cell proliferation and cell migration. **a** Representative pictures of colonies of cells transfected with shFASN or FASN overexpression plasmids. **b** Quantitation of cell colony numbers of 143B and MG-63 cells. **c** Quantitation of cell colony numbers of hFOB 1.19 and Saos-2 cells. **d** Transwell cell migration assay of 143B and MG-63 transfected with shFASN lentivirus and their controls. **e** Transwell cell migration assay of hFOB 1.19 and Saos-2 transfected with lentivirus containing FASN overexpression plasmids and their controls

**Fig. 4:**
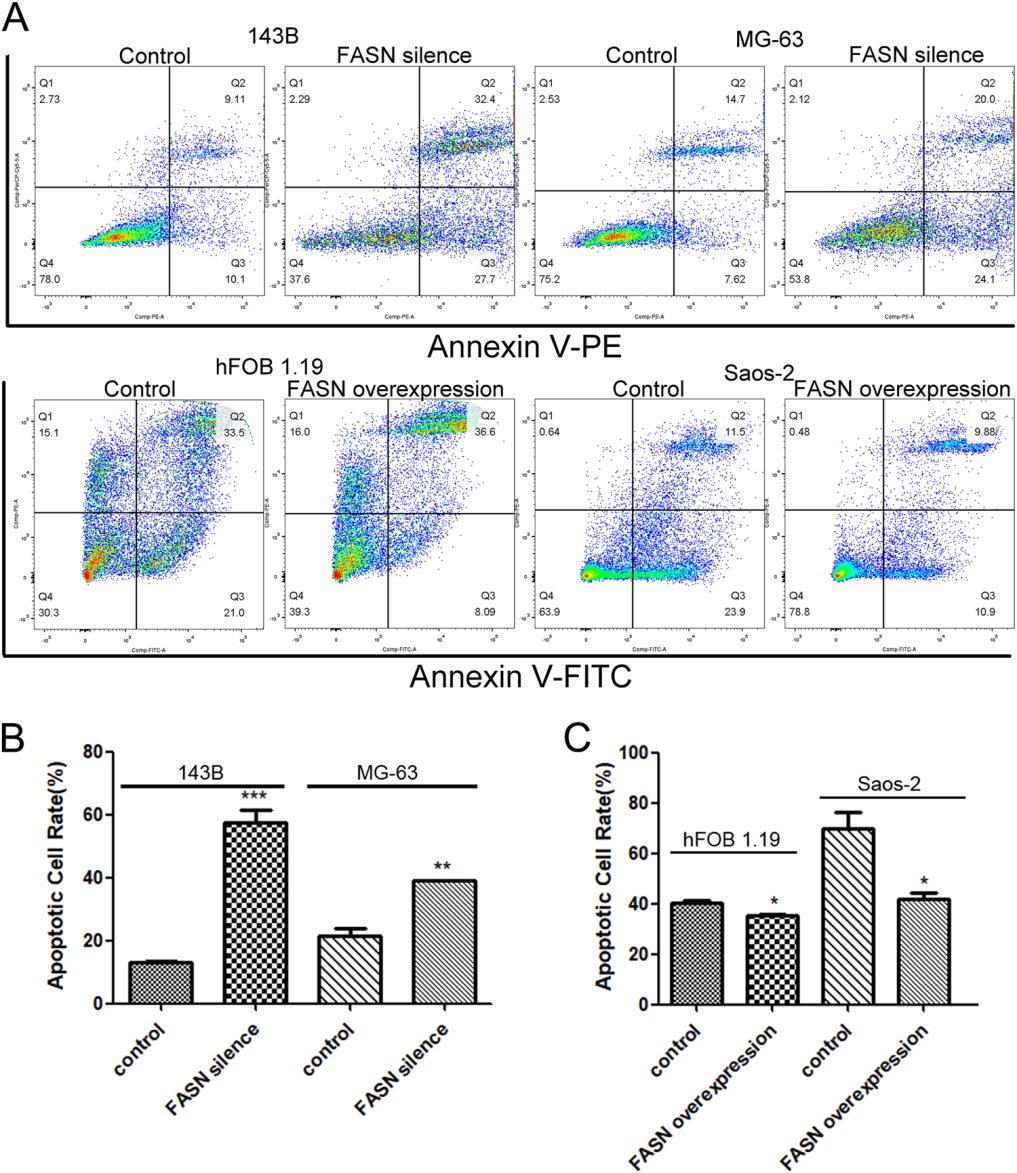
FASN inhibited cell apoptosis. **a** Apoptotic indexes of vector-transfected, shFASN-transfected or FASN-transfected cells were detected by fluorescence-activated cell sorting-based Annexin V double staining after suspension for two days. **b** Quantitation of apoptotic 143B and MG-63 cells. **c** Quantitation of apoptotic hFOB 1.19 and Saos-2 cells

We then studied the functions of FASN in suspended cells. Silence of FASN reduced cell colonies while overexpression of FASN promoted cell proliferation (Fig. 3a–c). To confirm FASN reduced cell number, we silenced FASN by another method (siRNA), and silence of FASN accelerated the cell death caused by suspension of cells (Fig S3A). Loss function of FASN also leaded to reduced cell colony number in suspended 143B and MG-63 (Fig S3B and Fig S3C). To confirm the results of silence RNA we constructed lentivirus silencing FASN and results showed that colony formation ability reduced after silencing FASN in 143B-AR cells (Fig S3D and Fig S3E). Loss of function of FASN inhibited cell migration while gain of function of FASN promoted cell migration (Fig. 3d, e). shFASN resulted in both early and total apoptotic 143B and MG-63 cells as determined by flow cytometry (Fig. 4a). In contrast, overexpression of FASN suppressed cell apoptosis (Fig. 4a). Statistical analysis also showed that the apoptotic cell rates were increased in FASN silence group (Fig. 4b) in suspended cells. In contrast, the apoptotic cell rates were decreased in FASN overexpression group (Fig. 4c). Silence of FASN also induced cell apoptosis in 143B-AR cells (Fig S4).

### FASN suppressed cell apoptosis via p-ERK1/2/Bcl-xL in suspended cells

**Fig. 5:**
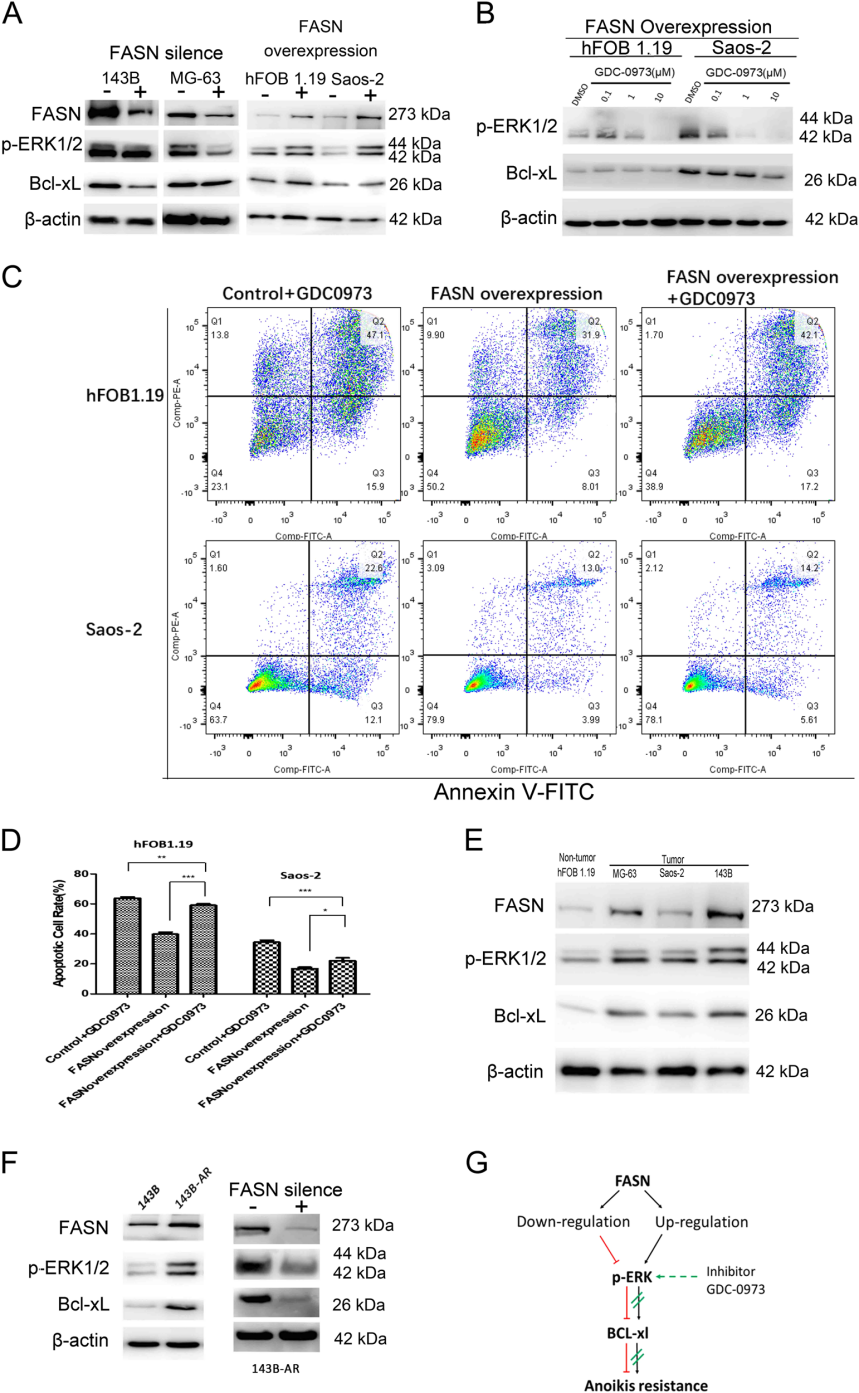
FASN suppressed cell apoptosis via ERK1/2/Bcl-xL in suspended cells. **a** Expression of FASN, p-ERK1/2 and Bcl-xL in stable cell lines silencing FASN. Expression of FASN, p-ERK1/2, and Bcl-xL in stable cell lines overexpressing FASN. **b** Expression of p-ERK1/2 and Bcl-xL after the stable cell lines overexpressing FASN were treated with ERK1/2 inhibitor GDC-0973. **c** Apoptotic indexes of cells treated with GDC-0973 (10 μM) were detected by Annexin V double staining. The apoptotic cells (Q3 and Q2) were more in the GDC-0973 treated FASN overexpression hFOB 1.19 and Saos-2 cells. The apoptotic cells (Q3 and Q2) were less in the GDC-0973 treated FASN overexpression hFOB 1.19 and Saos-2 cells compared with their controls. **d** Quantitation of apoptotic hFOB 1.19 and Saos-2 cells. **e** Expression of FASN, p-ERK1/2 and Bcl-xL in four kinds of osteosarcoma cells. **f** (Left) Relative expression levels of FASN in 143B and 143B-AR cells; (Right) Relative expression levels of FASN, p-ERK1/2 and Bcl-xL in 143B-AR cells. **g** Flow chart of summary of the relationships of FASN, p-ERK1/2, and Bcl-xL

We then tried to study the molecular pathway of this phenomenon and detected the expression of FASN and its potential related proteins p-ERK1/2^20^ and B-cell lymphoma-extra large (Bcl-xL)^21^. In addition, FASN was reported to regulate ERK1/2 in breast cancer^22^. ERK was reported to be relating to metastasis^23^. Thus, we tried to study whether FASN exerts it functions by regulating ERK1/2 and Bcl-xL in OS. We found the levels of p-ERK1/2 and Bcl-xL declined when FASN was silenced while they increased when FASN was overexpressed (Fig. 5a). In other words, the expression of p-ERK1/2 and Bcl-xL were consistent with FASN and they seemed to be regulated by FASN. We then used a compound called GDC-0973 (Cobimetinib)^24^ to specifically inhibit the activity of ERK1/2, results showed the upregulated Bcl-xL caused by overexpression of FASN were abolished by ERK1/2 inhibitors (Fig. 5b). To confirm the effects of GDC-0973 on the cellular levels, we conducted cell apoptosis assay (Fig. 5c). Because the GDC-0973 decreased the levels of anti-apoptotic molecule Bcl-xL (Fig. 5b), the GDC-0973 induced cell apoptosis in the FASN overexpression hFOB 1.19 and Saos-2 cells (Fig. 5c, d). FASN overexpression rescued the cell apoptosis rate caused by GDC-0973 (Fig. 5c, d). In other words, the effects of FASN promoting AR were weakened by ERK1/2 inhibitors. In addition, we found expression of these three molecules (FASN/ERK1/2/Bcl-xL) were consistent and increased simultaneously in tumor cells (Fig. 5e). In addition, results showed that the levels of FASN and its potential related molecules (p-ERK1/2 and Bcl-xL) increased in 143B-AR cells (Fig. 5f). The similar results happened in MG-63-AR cells (Fig S5B). When we silenced FASN in the 143B-AR cell, the p-ERK1/2 and Bcl-xL axes were also silenced (Fig. 5f). Then we used another method (siRNA) to silence FASN to confirm the results, siFASN resulted in decreased p-ERK1/2 in the suspended cells (Fig S5A). The proliferating cell nuclear antigen (PCNA) also increased (Fig S5B), which supported our previous reports that cell proliferation ability was higher in 143-AR cells (Fig. 1f). Taken together, all these results suggested that FASN increased the activity of p-ERK1/2/Bcl-xL, and FASN/p-ERK1/2/Bcl-xL pathway played vital roles in tumor and anoikis resistant (Fig. 5g).

### Inhibition of FASN in AR cells suppressed the lung metastasis of osteosarcoma

**Fig. 6:**
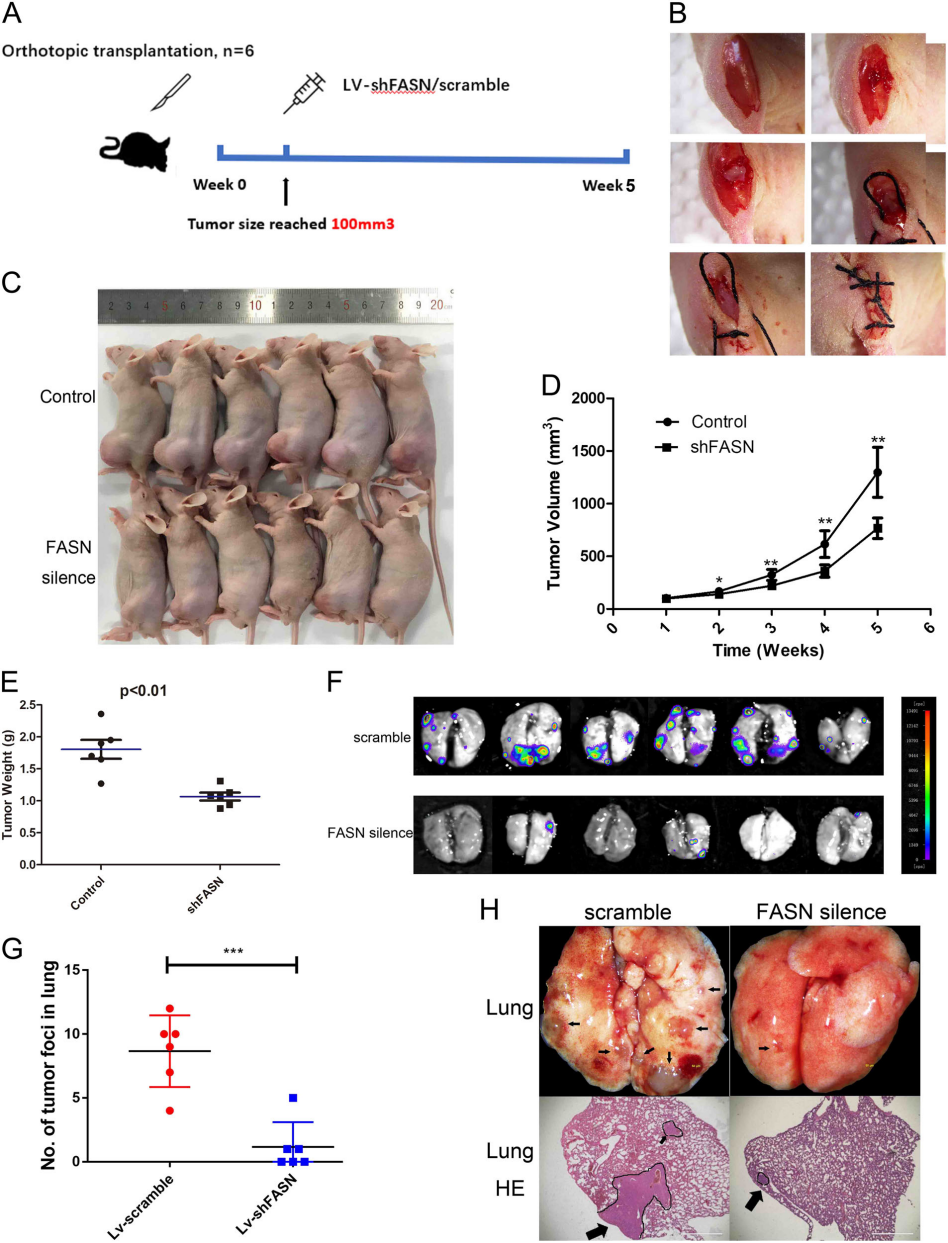
Inhibition of FASN suppressed the lung metastasis of osteosarcoma. **a** Flow chart of procedures of the study of the lung metastases of osteosarcoma in vivo. **b** Surgery steps of orthotopic transplantation. **c** The tumor size of mice treated with shFANS and controls. Representative examples of tumors formed in nude mice injected with the indicated cells. **d** Tumor growth curves were summarized in the line chart. **e** Tumor weight was summarized in the chart. **f** Lung tumors from osteosarcoma. **g** Number of tumor foci in lung of shFASN lentivirus-treated group and their control scramble group. **h** Tumor foci in lung and HE staining of the tumor. Arrows indicated the tumors. The average tumor volume was expressed as the mean ± SD of six mice. *P < 0.05; **P < 0.01

We then further confirmed our studies in vivo (Fig. 6a). The tumors were orthotopically transplanted into the nude mice (Fig. 6b). The tumor sizes of shFASN-treated group were smaller than the control group (Fig. 6c). The average volume of tumors induced by shFASN-143B cells was significantly smaller than that induced by Vector-143B cells (*P* < 0.05, Fig. 6d). In addition, the weight of tumor induced by shFASN-transfected cells was obviously lower than that induced by shControl-transfected cells (Fig. 6e). Moreover, silence of FASN inhibited the lung metastasis of osteosarcoma (Fig. 6f). The number of tumor foci in lung was significantly lower in the shFASN group (Fig. 6g). HE staining confirmed that there were smaller and less tumors in lung of mice treated with lentiviruses containing shFASN (Fig. 6h).

## Discussion

In the present study, we assume that FASN may prevent anoikis and promote metastasis in OS cells. We established anoikis resistant 143B cells by suspending common 143B cells and investigated the effects of anoikis resistant. We further studied the functions of FASN. We investigated the functions and potential molecular mechanisms of FASN in OS AR cells. The results revealed that FASN had higher expression levels in the OS cell lines 143B and MG-63 while lower expression in Saos-2 and hFOB 1.19, and suspension culture of adherent cells led to an increased expression levels of FASN. In addition, upregulation of FASN significantly augmented the anoikis resistance of cells and a knockdown of FASN significantly increased the rate of anoikis. In summary, FASN played important roles in OS cell proliferation, migration, and apoptosis in vitro and in vivo. We also studied the molecular mechanisms regulated by FASN. In these processes, FASN regulated the activity of ERK1/2/Bcl-xL. Furthermore, knockdown of FASN in suspension cells inhibited cells growth and pulmonary metastasis in nude mice.

In this study, we suspended four skeletal cell lines and found 143B had most strong ability of anoikis resistant. AR promoted OS cell proliferation and cell migration in vitro, and tumor growth in vivo. We constructed lentivirus silencing or overexpressing FASN and verified them by qPCR, western blot and ICC. By the opposite methods, we revealed that FASN promoted cell proliferation and migration and induced cell apoptosis via p-ERK1/2/Bcl-xL in suspended OS cells. Then we verified our results in vivo.

The length of mRNA of FASN is about 8.4 kb. Thus, clone full length is difficult. Overexpression of FASN is hard, so few previous studies reported overexpression of FASN. Some studies used transcription factor NF‑YA and they revealed that NF‑YA upregulated FASN^25^. But this should belong to an indirect way to upregulate FASN in cells. In this study, we used a human FASN cDNA ORF (NCBI Ref Seq BC007909) to construct the plasmids overexpressing FASN. We verified the expression of FASN in the transfected cells and demonstrated it functioned as an oncogene in suspended OS cells.

Our present study showed normal cells hFOB 1.19 died more than the other three tumor cells, indicating that tumor cells were more resistant to suspension causing cell death, which was one reason why tumor cells were more aggressive. Tumor cells tended to huddle together to resist to disadvantages including suspension and then survive when they leaved from their primary sites.

Our results showed the expression of FASN, p-ERK1/2, and Bcl-xL were consistent. In addition, the levels of p-ERK1/2 and Bcl-xL were consistent with the levels of FASN manipulated by us, suggesting that FASN could regulate the p-ERK1/2 and Bcl-xL. What’s more, we used ERK1/2 inhibitors and found the Bcl-xL was suppressed. These data were consistent with previous studies^26^. Taken together, all these results suggested FASN/ERK1/2/Bcl-xL was an important signaling pathway in the cancer.

We have reported that FASN inhibited OS cell invasion and migration via PI3K/Akt pathway^27^. In this study, we tried to look for alternative potential pathways regulated by FASN and found that ERK1/2/Bcl-xL might be another pathway. Our previous studies also showed that FASN was targeted by miRNA-424 which inhibited OS cell migration and invasion^28^. Other potential miRNAs regulating FASN could be further studied in the future.

microRNAs (miRNAs) are short noncoding RNAs that have substantial functions in cells, including cancer cells^29,30^. For instance, miRNAs activated osteogenesis^31^ or were related to functions of osteoclasts^32^. Besides cell level, miRNAs also influence molecular levels, including downregulating their targets. For example, miR-375 inhibited osteogenesis by targeting β-catenin of Wnt signaling pathways^33^. Besides normal bone cells, miRNAs also affect malignant phenotypes of the osteosarcoma. For instance, we reported that let-7g suppressed OS by targeting Aurora-B^34^. In other tumors, miRNAs also played vital roles, including metastasis of cancer^35^. Our further work should focus on exploring the miRNAs regulating FASN and the effects and functions of these miRNAs in regulating osteosarcoma.

## Materials and methods

### Cell culture

Osteosarcoma cell lines 143B, MG-63, Saos-2, and U2-OS and non-tumor control cell line hFOB 1.19 were cultured in DMEM, EMEM, McCOY’S 5A, DMEM, and DMEM/F12, respectively.

### Anoikis resistant cell survival rate assay

The same number of cells were forced to be suspended in the Costar ultra-low attachment 6-well plates (Corning) for 0, 2, 4, or 6 days. Then the cells were stained with Trypan Blue and counted.

### Clonogenic survival assay

After the culture of hFOB 1.19, Saos-2, MG-63, and 143B cells in forced suspension in Costar ultra-low attachment 6-well plates (Corning), cells were then plated into multiple well plates and incubated until colonies were formed. Then the cells were stained with crystal violet and counted.

### Western blot

The lysis buffer was RIPA buffer with protease inhibitor cocktail. The cells were added to lysis buffer and then underwent an alternant vortex and stored at 4 °C for half an hour. Then the cell lysis was centrifuged at 16,000 × *g* for 20 min at 4 °C. The Laemmli buffer was used to denature the samples. TGX™ FastCast™ Acrylamide Kit (Bio-rad), APS, and TEMED were used to make the gels. The PVDF membrane was blocked by 5% BSA solution for 1 h. Primary (first) antibodies included Caspase-3 antibody (Cell Signaling Technology, #9662), anti-fatty acid synthase antibody (Abcam, ab22759), Erk1/2 (Thr202/Tyr204) antibody (Cell Signaling Technology, #9101S), and anti-Bcl-xL antibody [E18] (Abcam, ab32370). The membrane was incubated with the primary antibody overnight at 4 °C, rinsed with TBST, and then incubated with the second antibody for 1 h at room temperature. ECL Prime western blotting reagents (GE Healthcare) were used to develop. The developer was myECL Imager (Thermo Fisher Scientific).

### ICC and immunofluorescence (IF)

We seeded cells into 12 wells chamber slices (ibidi). The cells were fixed with 4% PFA, permeabilized with 0.1% Triton X-100, blocked with 10% normal goat serum (Life Technologies), incubated with primary antibody overnight, and then secondary antibody Goat Anti-Rabbit Ig G (Alexa Fluor 488) (Abcam) at a 1/200 dilution.

### Transfection of FASN silence RNA

For transfection of silence RNA (siFASN), we added HiPerfect (Qiagen) transfection reagent to the medium without serum and gave a final HiPerFect concentration of 0.5% (v/v) after adding cells. We diluted siFASN in aforementioned medium, mixed, incubated for 10 min at room temperature, and added the cells.

### Lentiviruses silencing or overexpressing FASN

For shFASN (human), we firstly designed three shFASN and their sequences were shFASN1: GGTATGCGACGGGAAAGTATCCTCGAGGATACTTTCCCGTCGCATACC, shFASN2: ACATGGTCCTGAGCAGCTTTGCTCGAGCAAAGCTGCTCAGGACCATGT, shFASN3: CCTGCGTGGCCTTTGAAATGTCTCGAGACATTTCAAAGGCCACGCAGG. The inserts were ligated into the vector pLV-EGFP:T2A:Puro-EF1A. Western blot showed that shFASN3 had best inhibitory effects of FASN. Thus, we used shFASN3 in our further studies. For overexpressing FASN, we cloned the whole ORF of human FASN (NCBI Ref Seq: BC007909) into the overexpression vector pLVX-mCMV-mCherry-SV40-Hygro. The sequencing of whole cloned sequences was used to confirm the right sequences.

### Quantitative real-time PCR (qPCR)

TRIzol method was used for RNA extraction. The same amount of RNA was used for all the samples. Reverse transcription was performed by using SuperScript™ VILO™ MasterMix (Invitrogen) and random primer was used. Diluted cDNA was used for qPCR. The master mix was SYBR Green (Applied Biosystems). Each cDNA sample was triplicate in 96-well plate. Data were analyzed using the 2-ΔΔCT relative quantification method. The sequences of primers were listed in the Table 1. All the primers were verified by Primer-BLAST (NCBI). GAPDH was the internal control.

**Table 1:** Sequences of all primers

Primer name	Sequences (5′–3′)
GAPDH (homo sapiens) Forward	ATTGTCAGCAATGCATCCTG
GAPDH (homo sapiens) Reverse	ATGGACTGTGGTCATGAGCC
FASN (homo sapiens) Forward	CAACTCACGCTCCGGAAA
FASN (homo sapiens) Reverse	TGTGGATGCTGTCAAGGG

### Colony formation assay, wound healing assay, and transwell assay

They were described in our previous work^36–38^.

### Flow cytometry

The four stable cell lines silencing or overexpressing FASN were suspended in Costar® ultra-low attachment 6-well plates (Corning) for 2 days. The PE Annexin V Apoptosis Detection Kit with 7-AAD (Biolegend) and FITC Annexin V Apoptosis Detection Kit with PI were used.

### In vivo study

The animal ethic was approved by the Institutional Animal Care and Use Committee of Nanchang University. For the tumor growth animal model, 143B cells labeled with firefly luciferase were cultured as previously described to gain the ability of anoikis resistant. Approximately 2 × 10^7^ Luc-143B and AR-Luc-143B cells (suspended in 100 µl DMEM) were subcutaneously injected into the right flank of 4~ 6-week-old female Balb/c nude mice (*n* = 12). Tumor size was measured by caliper every week, and tumor volume was calculated using the following formula: volume = (*L* × *W*2/2). Tumors were harvested and weighted 5 weeks later, and tissues were frozen in liquid nitrigen for further detection.

For orthotopic osteosarcoma spontaneous metastasis animal model, the subcutaneously grown tumors of AR-Luc-143B were harvested and cut into small fragments (2–3 mm), a single fragment was transplanted into the left tibia of anesthetized nude mice and covered with bone wax (Braun). When the tumor size reached 100 mm^3^, mice were randomly assigned to two groups (*n* = 6), the lentivirus containing shFASN or their scramble controls (100 µl, 10^8^ PFU) were intratumorally injected. Mice were sacrificed 5 weeks after injection, and tumors were dissected and weighed. The lung tissues were imaged using the Night OWL LB 983 Imaging System (Berthold) to detect the pulmonary metastasis and fixed in 10% formalin for further study. Metastatic foci of lung were counted by ImageJ according to the bioluminescence imaging.

### Statistical analysis

Statistical analysis was performed by a *t*-test, and *P* < 0.05 was considered statistically significant.

## Supplementary information

Supplementary Figures

## Data Availability

The data supporting the results reported in the article can be found in supplementary figures.
