# Supplementary material for: Anoikis resistant mediated by FASN promoted growth and metastasis of osteosarcoma
Source: Cell Death Dis. 2019 Apr 1;10(4):298. doi: 10.1038/s41419-019-1532-2 (PMC6443797; doi:10.1038/s41419-019-1532-2)

**Supplementary Information**

Supplementary Figure Legend

**Fig. S1** Confirmation of successful establishment of stable cell lines silencing or overexpressing FASN by ICC. (**A**) Representative ICC staining images of cells. The intensity and the degree of green represented the expression levels of FASN. The 143B cells were less green in FASN silenced cells than controls. In other words, the degree of green was higher in the control cells than the cells with shFASN. (**B**) The MG-63 cells were less green in FASN silenced cells than controls. In other words, the degree of green was higher in the MG-63 control cells than the cells with shFASN. (**C**) The hFOB 1.19 cells were greener in FASN overexpression cells than controls. In other words, the degree of green was higher in the FASN overexpression cells than the controls.

**Fig. S2** Confirmation of successful establishment of stable cell lines overexpressing FASN. (**A**) The Saos-2 cells were greener in FASN overexpression cells than controls. In other words, the degree of green was higher in the FASN overexpression cells than the controls. (**B**) The magnification (10×) of Fig S1C.

**Fig. S3** FASN promoted proliferation in anoikis resistant cells. (**A**) Cell survival rate of 143B and MG-63 transfected with siFASN. (**B**) 143B and MG-63 were transfected with siFASN and suspended, and then proceeded to colony formation assay. Representative pictures of colonies of 143B and MG-63 cells. (**C**) Quantitation of cell colony numbers. (**D**) 143B-AR were transfected with shFASN and then proceeded to colony formation assay. Representative pictures of colonies. (**E**) Quantitation of cell colony numbers.

**Fig. S4** FASN inhibited cell apoptosis in anoikis resistant cells. (**A**) Apoptotic indexes of vector-transfected, shFASN-transfected 143B-AR cells were detected by Annexin V double staining. (**B**) Quantitation of apoptotic cells. * p < 0.05, ** p < 0.01, n = 3/group.

**Fig. S5** FASN increased in anoikis resistant cells. (**A**) Relative expression levels of FASN in AR and their control cells. (**B**) 143B and MG-63 were transfected with siFASN and suspended, and then proceeded to Western Blot. Relative expression levels of p-ERK1/2 in 143B and MG-63 cells.

Supplementary Figures


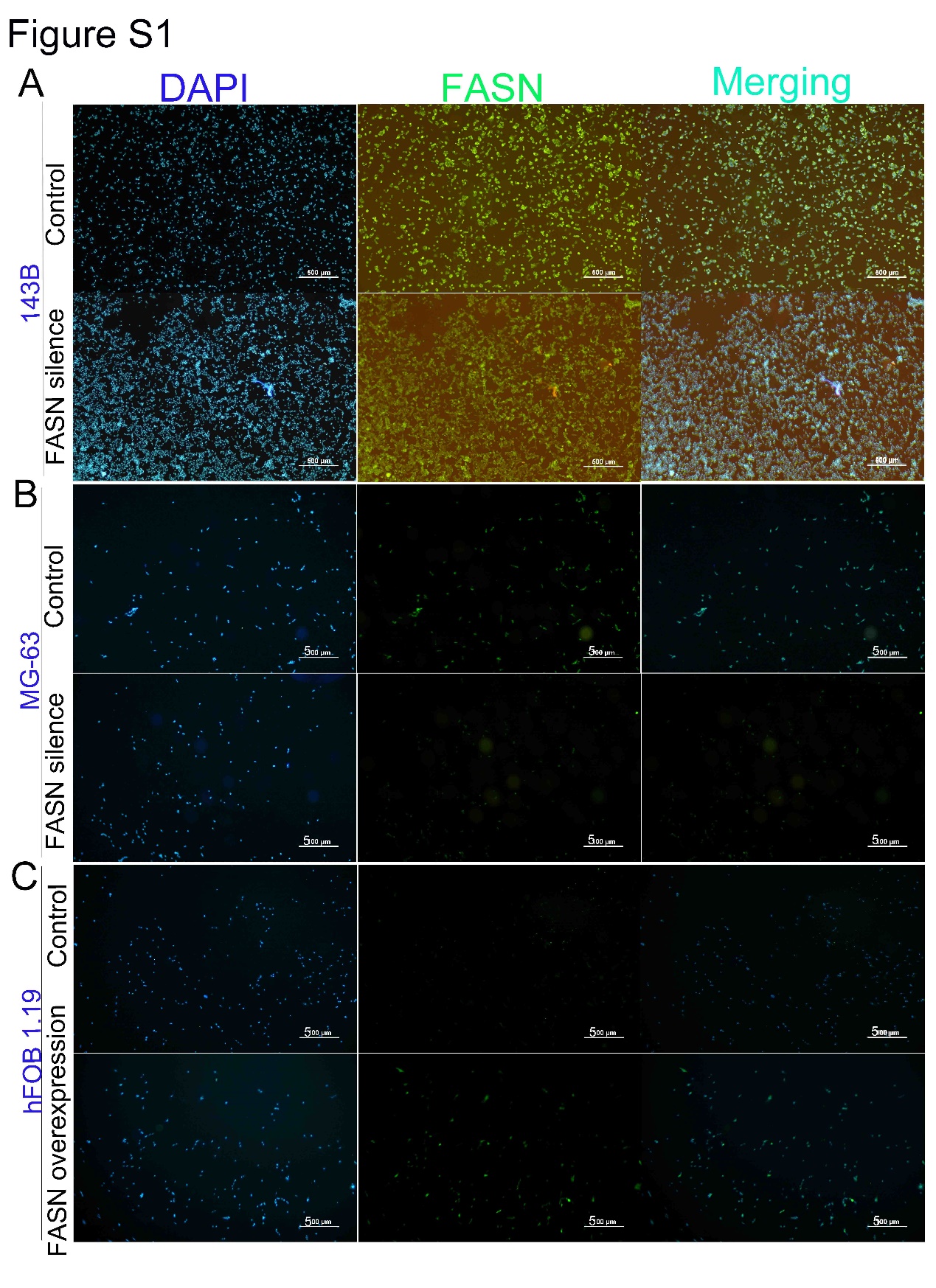


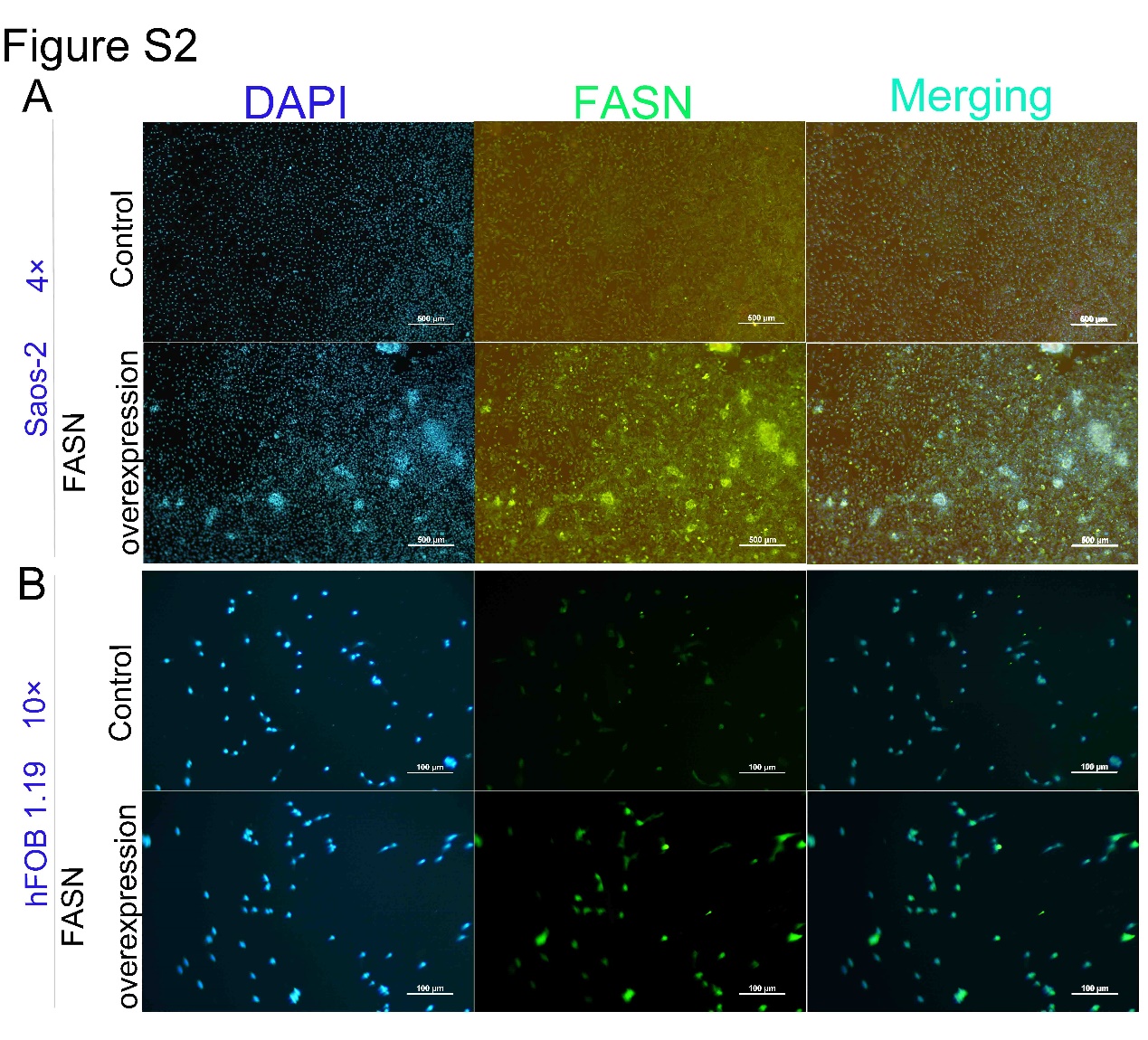


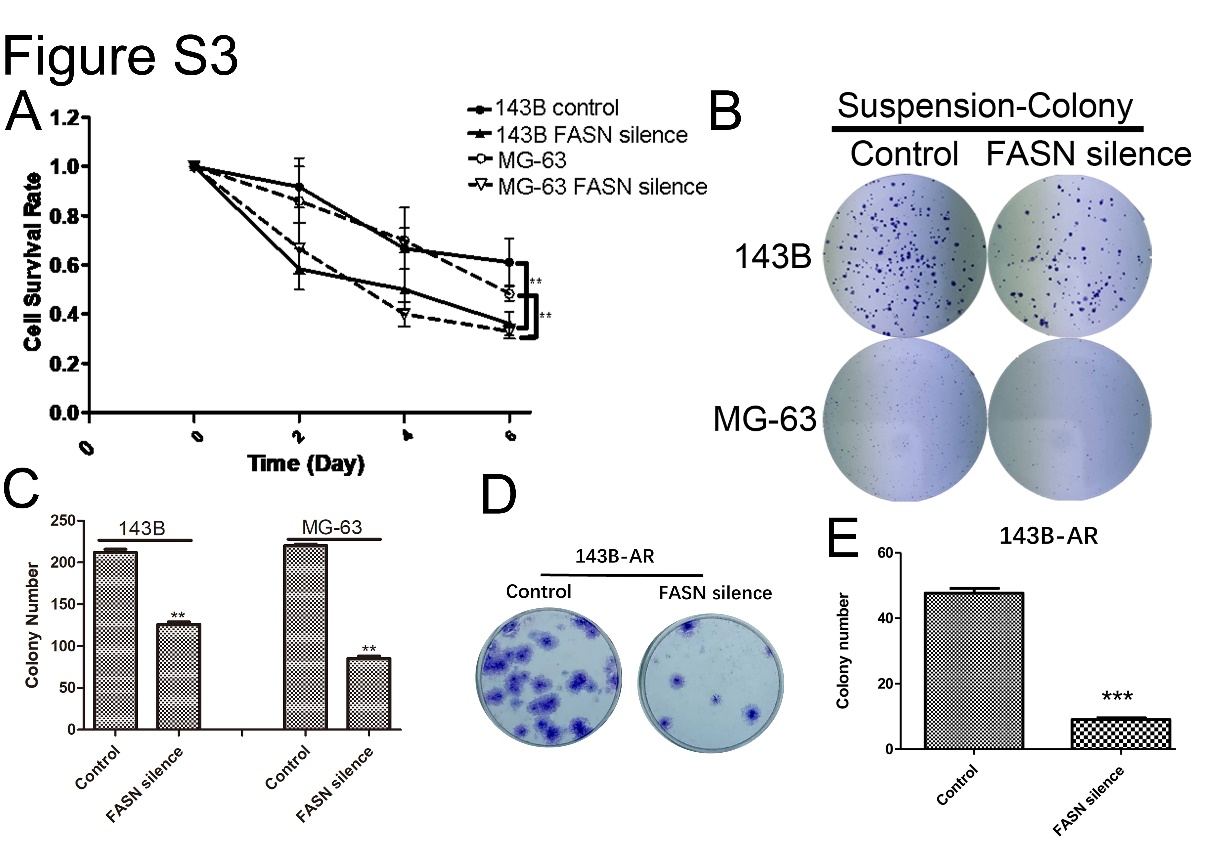


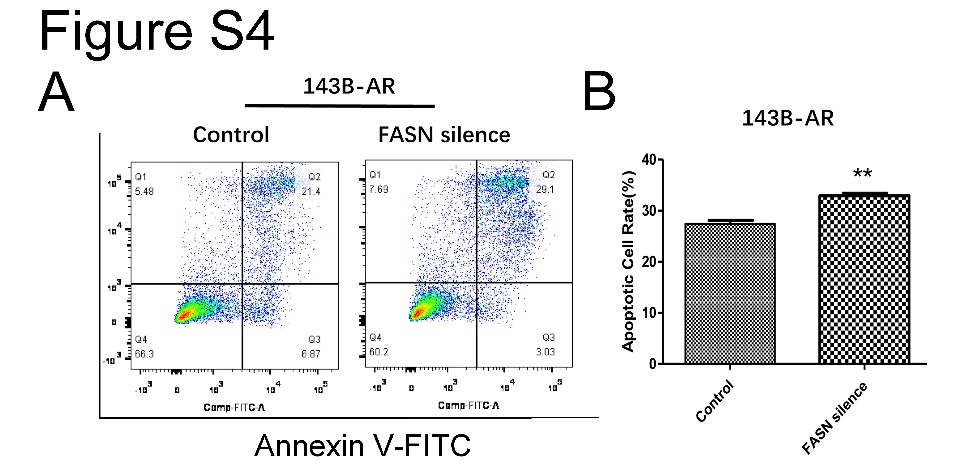


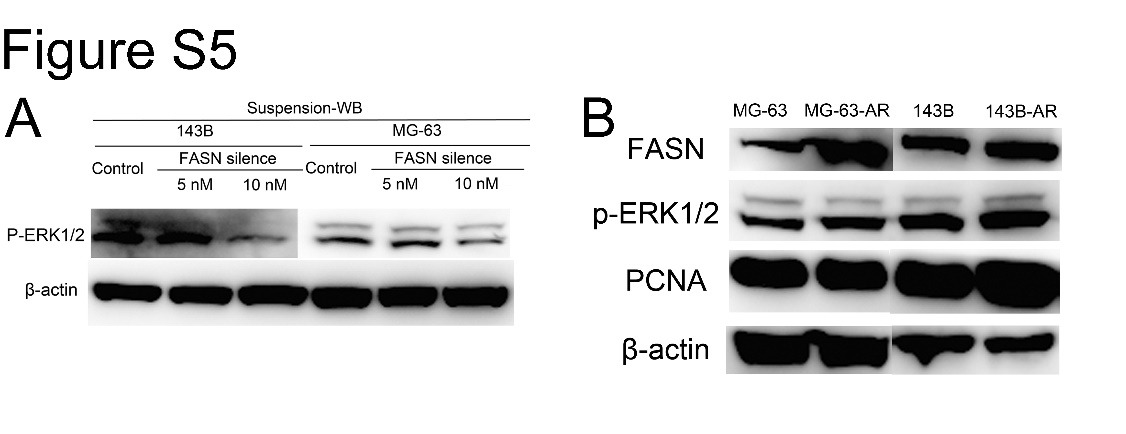

Supplement: Supplementary file 1 — Supplementary Figures [file 41419_2019_1532_MOESM1_ESM.docx]
